# High-throughput plant breeding approaches: Moving along with plant-based food demands for pet food industries

**DOI:** 10.3389/fvets.2022.991844

**Published:** 2022-09-30

**Authors:** Mohsen Yoosefzadeh-Najafabadi, Istvan Rajcan, Mahsa Vazin

**Affiliations:** ^1^Department of Plant Agriculture, University of Guelph, Guelph, ON, Canada; ^2^PawCo Foods, San Francisco, CA, United States

**Keywords:** cultivar development, COVID-19, high-throughput, meatless, multi-traits, multi-omics

## Introduction

**Figure 1:**
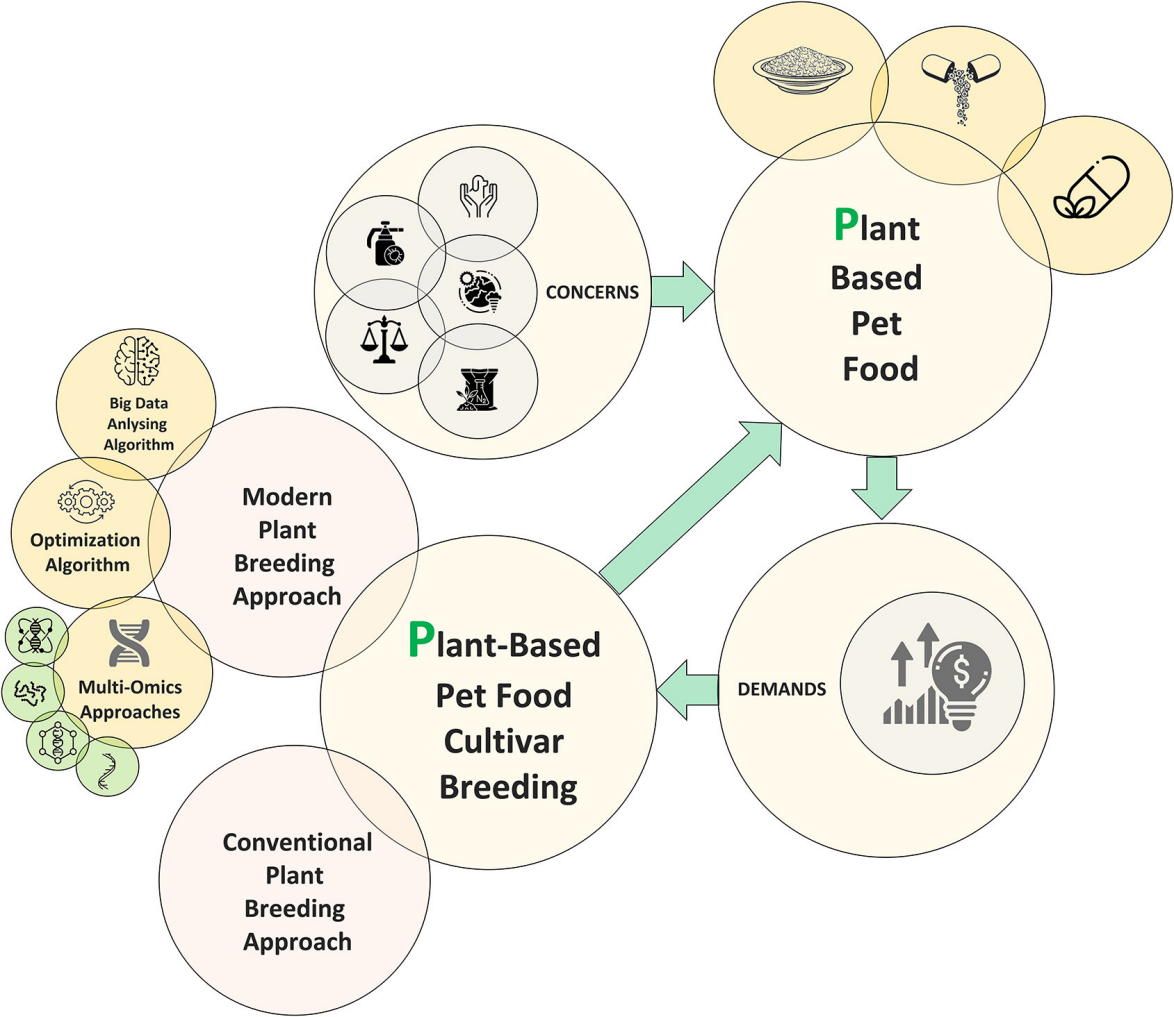
Schematic view of plant cultivar breeding pipeline for plant-based pet food production.

Moving toward consuming plant-based meat while shifting away from conventional meat may benefit the environment, animal welfare, and human health (1). For many decades, conventional plant-based proteins such as seitan, tempeh, and tofu, as well as other vegetarian meat-like foods, have been commercially accessible (2). However, plant-based food demands have significantly increased over the last decades, especially in Western countries (3). The high demands for plant-based foods are not only limited to humans, as many companion animal owners are continuously concerned about the possible correlations between the consumption of animal products, even for their pets, and animal welfare problems, herbicides and fertilizers, degenerative health conditions, and climate change (Figure 1) (4).

In the United States, cats and dogs outnumber children under the age of 18 by nearly two to one, with 83 and 95 million pet dogs and cats, respectively (5). In Europe, especially in the United Kingdom, more than 57 million pets have been spread across ~40% of households, and the pet population is increasing by one percent annually (6). Several studies have been done all around Europe to find the possible reasons for changing the current common pet diet to plant-based, and the results indicated that animal welfare, ethical, and moral concerns are the three most important concerns that make pet owners willingly select the plant-based diet for their pets (4, 7–9). Therefore, efficient and effective plant-based meat production is now a high trend in pet industries in order to keep pace with high plant-based meat demands in the near future.

Meat has long been seen as a vital part of a balanced diet since it contains many valuable nutrients, such as high biological value protein, iron, vitamin B12, other B complex vitamins, zinc, selenium, and phosphorus (10). In addition, meat is considered as an important dietary component due to its high protein content and complete amino acid profile. Twenty different amino acids, as building blocks of proteins, are used to construct proteins; eight are considered essential for humans (10 for dogs), meaning they must be ingested as food as the body does not produce them (10). The remaining amino acids can be created in the body and are hence considered non-essential. However, it would be necessary to supply the required raw components for the body to produce the non-essential amino acids sufficiently.

Different plant protein sources, such as soybean, which contain all essential amino acids (11), can be used as textured vegetable protein (TVP), an alternative to meat consumption. Nonetheless, TVP made from soybeans lacks important nutrients such as low levels of tryptophan, methionine, vitamin B12, and vitamin D that meat provides sufficiently. In addition, there are some nutrients from TVP sources that may not be well absorbed in the body (12). Therefore, additional nutrients and/ or various plant protein sources should be embedded/adjusted into TVP to be considered as nutritionally valuable/complete as meat for plant-based food diets (Figure 1).

While the plant-based food demands for pets are increasing continuously, it would be necessary to develop plant cultivars that require less adjustment for optimal pet foods. The typical plant breeding process, from crossing to releasing a cultivar, will take many years (e.g., at least 10 years in soybean) (13). In addition, breeding for complex traits that are under control by several genetic and environmental factors even makes the breeding process cumbersome, expensive, and time-consuming (14). However, the recent advances in high throughput approaches can facilitate the plant breeding process by measuring several traits in a less expensive and timely manner, making the breeding decision more accurate and shortening the breeding cycle (15).

Here in this opinion paper, we briefly explain the current status/progress of plant-based pet foods, describe the high throughput plant breeding approach, elaborate on the recent high throughput approaches in plant breeding programs, and finally provide a vision for possible financial investment in developing cultivars for plant-based food industries.

## Consume meats to meet nutritional demands: Still valid for today's life?

Throughout history, humans have always consumed meats to meet nutritional demands. However, meat consumption has increased dramatically over the last century, despite the availability of non-meat-based foods (16). Therefore, meat production has expanded fourfold since the 1960s to fulfill these rising demands, outpacing population growth. As a result, by 2050, worldwide meat consumption is expected to treble from 2008 despite the negative impacts that can be made on environmental sustainability, human health, and ethical considerations, improving public health and minimizing animal suffering (17). The main question here is whether consuming meats is still required to meet nutritional demands?

Plant foods such as soybeans, peas, beans, chickpeas, peanuts, and lentils have a long history of being used as protein sources in a broad range of cultural and international cuisines. Many are excellent protein sources, but few contain all the essential amino acids at a level that is sufficient to offer a full nutritional profile required for dogs. In addition, plant-based diets are not offering taurine, which is essential for cats. Therefore, it should be synthetically added as a supplement to plant-based pet food. Consequently, various plant-based protein sources must be used to meet nutritional needs, while most animal protein, like meat or dairy, does the whole job by itself, being a complete protein (18). As an example, soy protein is one of the important plant-based protein sources containing all the essential amino acids (11). However, it has to be used in combination with other plant sources to offer a well-balanced dietary profile for dogs (Figure 1).

## Plant-based pet foods are not perfect for your pets: Rumor or real?

Animal food is not all about getting the optimum amount of protein every day but considering the amino acids that those proteins are formed of Knight and Leitsberger (4). Most pet food industries usually consider the amino acid profile as the priority to ensure that all of the required amino acids are included in the pet food (8). The key point of producing plant-based pet foods is to obtain the right combinations of different ingredients, such as plants, vitamins, and minerals (4). However, some believe that the current plant-based pet foods serve as double-edged swords with their merits and demerits and plant-based pet foods are underpowered to meet the criteria for carnivorous pets, such as cats (19). Dogs are known for being opportunistic feeder and can feed well on balanced plant-based pet foods, while cats are conventionally known as obligated carnivores, and meat is supposed to be included in their diets (19). However, a significant and growing body of population studies and case reports have indicated that both dogs and cats maintained on vegetarian diets may be healthy and, indeed, may experience a range of health benefits (4, 20).

## Plant-based pet food status in the post-COVID-19 pandemic era

As business shutdowns, travel restrictions, and lockdowns were the three main inevitable components of the COVID-19 pandemic, a significant number of businesses experienced a challenging time, especially the food and beverage industries (21). The closure of several facilities and companies is obstructing global supply chains and negatively affecting production processes, delivery schedules, and sales of various items (21). Although the value chain disruption during the early stages of the COVID-19 pandemic had negatively impacted the plant-based pet food market, most people added new pets to their homes due to the remote work situation (22). Therefore, the COVID-19 pandemic has not had a large detrimental influence on the market for plant-based pet food, while the demand for plant-based pet food online shopping increased significantly (Figure 1) (22). Based on research conducted by Insight Partners, the global market for plant-based pet food production is expected to increase from nearly US$8.6 billion in 2021 to almost US$15.6 billion by 2028, which clearly indicates the huge investment in this area (23).

## Cultivar development based on the pet food industries demands

One major problem that makes plant-based pet food production difficult is balancing the food based on several ingredients from different resources to ensure all nutritional needs are met (24). A single, complete protein source such as TVP is commonly used in plant-based pet food, which provides all the essential amino acids for dogs, but it has to be balanced for the right amount of essential amino acids to provide all the necessary nutrients at the required level that a meat-based diet offers (8). To face these challenges, several pet food companies provide dogs with all the nutrients they need by carefully formulating recipes based on a combination of yeast and plant proteins. The recipes are also supplemented with additional sources to ensure a complete and balanced vegan meal. However, adjusting plant-based pet foods is laborious and time-consuming, and pet food companies try to reduce their dependencies on several ingredients as much as possible. Here, the plant breeding approaches may be useful to breed for a well-balanced plant-based protein source. Instead of combining different plant protein sources, it would be convenient to develop a single resource that is equal to meat in terms of nutrition.

## High throughput approaches to facilitate plant breeding programs

Nowadays, with significant advancements in high-throughput approaches in different plant omics such as phenomics, genomics, transcriptomics, proteomics, epigenomics, and metabolomics, plant breeders are now able to speed up the breeding process and make decisions more accurate (Breeding era 4.0), especially for complex traits such as yield, protein, oil, and metabolite concentrations (25). As an example, increasing methionine concentration in soybean is a game changer parameter in plant-based pet food production which requires a comprehensive environment, genetic, and other omics information (Figure 1). The possible breeding pipeline to increase the methionine concentration in soybean is to (i) quantifying the amount of methionine using targeted approaches to select the superior genotypes that are high in methionine concentration, (ii) crossing the high methionine soybean genotype with high yield, high protein soybean cultivar, (iii) determining the genes and transcripts associate with the tested traits to detect the possible correlation between high yield, high protein and high methionine concentration genotypes using genome/transcriptome-wide association studies, (iii) detecting the possible linkage, epistasis, and/or pleiotropy effects existed with the loci governing methionine and other traits of interest, (iv) predicting the genotype performance using sophisticated bigdata analyzing methods to make the breeding decision more accurate, (v) speeding up the breeding progress by cultivating more than one generation in a year, (vi) making the breeding cycle short by using the associated genomic/transcriptomic regions with a trait of interest as markers, and (vii) using genome editing technologies in parallel with the conventional breeding methods to precisely modify several associated genomic regions with the tested traits per generation and add new value-added traits to the developed plant cultivar.

As several traits are required to be adjusted in developing cultivars for plant-based pet food production, the optimum values of the tested traits have to be determined based on their genetic relationship with other desirable traits (26). Nowadays, optimization algorithms (Figure 1), which optimize the input variables to maximize the value of the output variable, are implemented in plant breeding to improve multi-trait selection (26). In the methionine example, optimization algorithms can be employed considering the genotypic information of each genotype as input variables and methionine, protein, and yield as outputs. The outcome would be detecting the optimum condition of genomic regions in order to have a soybean cultivar with high yield, protein, and methionine concentration. Such results can be used in genome editing or/and marker-assisted selection pipelines to speed up the breeding process.

## Conclusion and future perspective

Due to animal welfare, ethical, moral, and climate change concerns around producing meat-based food, pet owners are willing to change their pets' diets by shifting to plant-based pet foods. As a result, plant-based pet food production grows exponentially, and there is a dire need to facilitate the speed of food production to keep pace with future demands. Meanwhile, reaching the optimum amount of protein/amino acid profiles in plant-based pet foods requires a precise combination of different plant-sourced ingredients as there are no individual plant sources that are sufficient to meet all plant-based pet food criteria. However, by introducing plant breeding cultivar development pipelines specifically for pet food industries, we could be able to reduce the number of plant-sourced ingredients in plant-based pet foods. This endeavor requires multi-trait plant breeding programs as several plant components need to be adjusted simultaneously, for plant-based pet food production, which requires precise use of optimization algorithms in this area. Meanwhile, to speed up the breeding process and shorten the breeding cycle, breeders may want to use high-throughput methods and sophisticated bigdata analyzing methods in their breeding programs. In addition, this opinion paper suggested that financial agents invest in plant breeding programs to develop high-quality plant cultivars to meet the future demands of plant-based pet food production. As the future direction, several important areas are still less explored and need further investigations in plant-based pet food production, such as (1) ethical concerns to assure that pet owners are not imposing their personal views on their pets, and (2) longevity and palatability of plant-based pet foods.

## Author contributions

All authors listed have made a substantial, direct, and intellectual contribution to the work and approved it for publication.

## Conflict of interest

Author MV was employed by PawCo Foods. The remaining authors declare that the research was conducted in the absence of any commercial or financial relationships that could be construed as a potential conflict of interest.

## Publisher's note

All claims expressed in this article are solely those of the authors and do not necessarily represent those of their affiliated organizations, or those of the publisher, the editors and the reviewers. Any product that may be evaluated in this article, or claim that may be made by its manufacturer, is not guaranteed or endorsed by the publisher.
